# Walking the Tightrope of Job Demands and Resources: Leveraging Work Engagement to Counter Turnover Intentions of Information Technology Professionals

**DOI:** 10.3389/fpsyg.2022.660308

**Published:** 2022-06-02

**Authors:** Jana Van Heerden, Marieta Du Plessis, Jurgen R. Becker

**Affiliations:** Department of Industrial Psychology, University of the Western Cape, Bellville, South Africa

**Keywords:** work engagement, turnover intention, information technology professionals, job demands, job resources, South Africa, banking industry, indirect effect

## Abstract

Organisations within the banking industry are increasingly confronted with attraction and retention challenges within their Information Technology (IT) divisions, driven by an increase in demand for skilled resources within the market. Therefore, the primary objective of the study was to explore the impact of job resources and job demands on work engagement and employee turnover intentions within the IT division of a South African bank. The Job Demands-Resources (JD-R) model was applied as theoretical framework to identify the unique job resources and job demands driving work engagement and turnover intentions of employees within this highly specialised section of the South African banking industry. Quantitative data was collected from 239 IT professionals *via* a self-administered, web-based survey measuring work engagement, job demands and resources, and turnover intentions. After confirmation of the factor structures of each of the variables, the direct and indirect relationships between the variables were analysed. The results indicate statistically significant relationships between job resources, work engagement and turnover intentions. Job demands moderated the relationship between job resources and work engagement, whilst work engagement mediated the relationship between job resources and turnover intention. By applying the JD-R model as a theoretical framework for the study, the unique job resources and job demands as drivers of work engagement and turnover intentions of IT employees could be highlighted to direct the development of focused work engagement and retention strategies.

## Introduction

In the agile global environment, organisational success greatly depends on an organisation’s ability to effectively apply Information Technology (IT), and to ensure the availability and performance of their IT employees. Specifically, the success of the banking industry, with the possibility of complete digitisation of their services, depends on the effective deployment of information technology. This has become even more prominent amidst the COVID-19 pandemic, where agile organisations that could innovate through adoption or adaptation of technology have been somewhat shielded of the negative business impacts of the pandemic (Seetharman, 2020). Even though most organisations consider their IT employees as key value-adding resources that form a significant part of the business, these individuals are regarded as indispensable in creating systems, processes and procedures to help organisations navigate the new normal (Arshad, 2020). Due to shortage in supply of IT employees they often have high bargaining power and tend to move between organisations who offer better career prospects and benefits. Organisations within the IT driven banking industry are challenged by a decrease in the availability of technically competent and skilled professionals due to the increase in demand for these skills (Koong et al., 2020). Competitors within the same industry will use aggressive recruitment techniques supported by various forms of monetary and non-monetary rewards in an attempt to attract the best talent. This supply-demand gap in the IT profession contributes to the staffing challenge: if IT professionals are not content within their current work environment, they are likely to find alternative employment opportunities in abundance. The loss of these resources could have a significant impact on delivery of key business objectives; reflected in disruptions in project flows, impact on deliverable quality, and loss of intellectual property.

Therefore, the main objective of the study was to gain a deeper understanding of the factors that drive employee tenure and satisfaction in IT positions. Due to the link between employee engagement levels and turnover intentions (i.e., Harter et al., 2002; Bakker et al., 2003a; Schaufeli and Bakker, 2004; Bakker et al., 2008), organisations should strive towards understanding the drivers of engagement to ensure effective retention strategies can be developed to retain these employees. During the March 2019 to December 2020 reporting period, the IT division reported an average voluntary turnover of 10.5% of the overall headcount. Taking the current supply and demand challenges associated with scarce and critical IT resources into consideration, organisations will have to gain an understanding of the factors influencing their employees’ levels of engagement and intention to stay with the organisation. One possible avenue of research suggest that broad categories of job resources and job demands may have a strong impact on work engagement. This research study was undertaken with a sample of IT professionals within the IT division of a South African bank to: (i) investigate job demands and job resources as factors impacting work engagement; (ii) determine which of the identified drivers of work engagement (job demands vs. job resources) have the most significant impact on turnover intention, (iii) determine the indirect effect of work engagement on job resources and turnover intentions, and (iv) provide recommendations to the organisation to assist with the development of a retention strategy to increase employee intention to stay with a specific focus on scarce and critical IT skills and resources.

### Literature Review

Work engagement is defined as “a positive, fulfilling work-related state of mind that is characterised by vigour, dedication, and absorption” (Schaufeli and Bakker, 2004, p. 295). It is not surprising that the work engagement construct and three supporting dimensions of vigour (high levels of energy and mental resilience during job performance, the willingness to invest effort in one’s work, not being easily drained, being persistent even in the face of adversity or challenge), dedication (ability to derive meaning or significance from work through enthusiasm, being proud of and feeling inspired and challenged by one’s work), and absorption (satisfactory state of complete immersion in work, characterised by focused attention, time distortion, a loss of self-consciousness, effortless concentration, complete control, and intrinsic gratification), is presumed to be a strictly positive and relatively stable indicator of occupational wellbeing (Schaufeli et al., 2002; Storm and Rothmann, 2003). Employees with high engagement levels would be willing to function beyond their core responsibilities outlined by a job description, apply innovative and “out-of-the-box” thinking in an effort to move their organisations forward (Markos and Sridevi, 2010), and will exhibit a stronger awareness of the business context and actively work with their colleagues to improve on-the-job performance for organisational benefit (Robinson et al., 2004). Engaged employees are emotionally attached to their organisation and highly involved in their jobs (Markos and Sridevi, 2010), exhibiting great eagerness to contribute to their employer’s success by going beyond their employment contract. These perspectives emphasize the importance for organisations providing employees with meaningful work that contributes to personal fulfilment and motivation (Coetzer and Rothmann, 2007), enabling increased employee motivation, growth, empowerment, and involvement (Spreitzer et al., 1997). Organisations should actively strive to develop and foster work engagement through a collaborative relationship between the employer and employee.

Demerouti et al. (2001) developed the JD-R model based on the assumption that two underlying psychological processes play a significant role in ensuring the welfare of individuals: an effort driven process in which disproportionate job demands (mental, emotional, and physical) and an absence of job resources (support, autonomy, and feedback) contribute to levels of distress, and a motivation-driven process in which job resources lead to work engagement. Balducci et al. (2011) recommend applying the JD-R model as a conceptual framework in all occupational settings to study the drivers associated with work engagement.

According to Van den Broeck et al. (2017), job demands are those physical, psychological, social, or organizational aspects of work that require sustained physical and/or psychological effort or skills and are energy-depleting., and could potentially contribute to strain in instances where the employee’s adaptive capability is surpassed (Rothmann et al., 2006). Job demands include the physical, social, organisational and situational aspects of a job (i.e., role ambiguity, role conflict, heavy workload, and work pressure) that require continued physical and/or psychological effort on the part of the employee, according to Rothmann (2003). When job demands are chronically high and the external environment presents a lack of required resources, the individuals are not able to reduce the potential negative influence of high job demands to achieve their work goals as their energy is progressively drained (Schaufeli, 2017).

According to Rothmann et al. (2006), overload as a job demand includes physical, cognitive, and emotional load related to time pressure (pace of work), attentiveness to many things at the same time (amount of work), mental and emotional load (dealing with power struggles), and job characteristics related to task interruptions, workload, work-home interferences, organisational changes, and emotional dissonance (Bakker et al., 2003b; Van den Broeck et al., 2008). Many of these demands are prevalent for IT professionals as employment in this industry is often intensive with ongoing or intermittent engagement beyond traditional working hours (DePaquale et al., 2017). Job demands are associated with high costs that elicit negative responses such as depression, anxiety, and burnout (Schaufeli and Bakker, 2004), leading to a subsequently decrease in employee engagement levels.

Bakker et al. (2008) regard job resources as key factors associated with employee engagement. Job resources are related to the extent to which the job offers opportunities to individual employees and include physical, psychological, social, or organisational aspects of the job that decreases job demands and related physiological and psychological costs, are practical in achieving work goals, and stimulate personal growth, learning, and development (Demerouti et al., 2001). Bakker (2011) further elaborated on the JD-R model by assuming that both job resources (i.e., autonomy, performance feedback, social support, and supervisory coaching) and personal resources (i.e., optimism, self-efficacy, resilience, and self-esteem) are strong predictors of work engagement (Bakker et al., 2008), especially in the presence of high job demands (i.e., work pressure, emotional demands, and physical demands) (Janse van Rensburg et al., 2013). Job and personal resources initiate a motivational process as highly engaged and performing employees are able to create their own resources to further foster engagement and improve their performance (Bakker, 2011). This process of actively altering or influencing their work environments and job characteristics is referred to as job crafting defined as the “self-initiated changes that employees make in their own job demands and job resources to attain and/or optimise their personal (work) goals” (Tims et al., 2012, p. 173). Job resources are also considered a crucial element for ensuring employee retention (De Braine and Roodt, 2011) with Rothmann and Jordaan (2006) reporting empirical evidence that job resources are able to shield individuals and organisations from the potential negative impact of job demands on burnout. De Lange et al. (2008) found that low levels of work engagement, job autonomy, and departmental resources predicted employees’ intentions to leave their employer and transferring to other companies, providing an indication of the employees’ turnover intentions.

A longitudinal study by Mauno et al. (2007) has highlighted job resources as better predictors of the levels of employee engagement than job demands. These job resources refer to aspects related to social support from colleagues and the intrinsic nature of the job, including skills variety, autonomy, and learning opportunities. It would, however, seem that individuals could experience work engagement despite higher work demands. In these instances, the availability of relevant and appropriate job resources could moderate the effect of job demands on the employees’ levels of engagement. Job resources could, therefore, diminish the effect of job demands on work engagement, according to Hakanen et al. (2005). This is due to a weak relationship between job demands and work engagement in individuals with high job resources. It is, therefore, proposed that job demands moderate the relationship between job resources and work engagement.

Hoonakker et al. (2013) proposed the use of the JD-R model as a theoretical basis for predicting turnover intentions by explaining the relationships between job demands and job stress, job resources and job satisfaction/commitment, and turnover intentions. Job and organisational design literature have revealed various job demands that are positively related to turnover intentions, including disproportionate workload (i.e., Houkes et al., 2003), role stressors associated with performing tasks not in the employee’s job description or role ambiguity (i.e., Asiwe et al., 2015), and a lack of challenge (i.e., Mathieu and Zajac, 1990) characterised by task repetitiveness and excessive routine (i.e., Griffeth et al., 2000). High levels of stress are prevalent in individuals experiencing work overload (Schaufeli and Bakker, 2004), characterised by feeling overwhelmed by perceived time pressures and deadlines, and information overload (Montgomery et al., 2003). As many IT related jobs expect of employees to work late, be on-call to address technical problems, and even travel extensively, all of these factors can contribute to conflict between work and family-life, leading to inter-role conflict that could occur in instances when the demands of work and family are equally incompatible (Greenhaus and Beutell, 1985). Employees are motivated by interesting and challenging work that offers them an opportunity to apply their skills and experience, and encourages learning opportunities and information exchange. IT professionals will constantly seek opportunities to work on projects in an effort to enhance their own career, knowledge, and future earning power. According to Ang and Slaughter (2001), job design characteristics have been found to impact employee attitudes, behaviours, and job performance, including job resources such as autonomy, job feedback from management, skills variety, job identity, and job significance.

**FIGURE 1:**
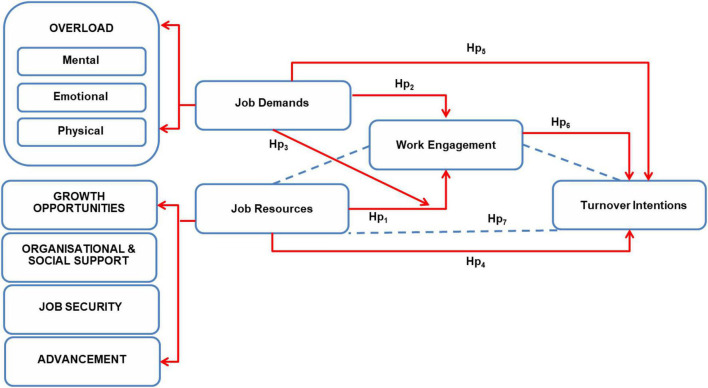
Theoretical model of the relationships between the variables. Hp, hypothesis.

Due to the link between engagement and retention, there is a lower probability of highly engaged employees leaving an organisation on a voluntary basis (Harter et al., 2002; Firth et al., 2004). Empirical studies (see Schaufeli and Bakker, 2004; Kim, 2017) provided empirical evidence of the link between work engagement and turnover intention as well as, the relationship between the absence of job resources and higher levels of disengagement, which increases turnover intentions. Job resources positively affected work engagement which, in turn, negatively predicted the turnover intention proposed by the motivational process. It is subsequently suggested that engagement is exclusively predicated by the availability of job resources, relates only to turnover intentions, and mediates the relationship between job resources and turnover intentions. The literature review informed the hypotheses or the quantitative study on a sample population of IT professionals within the banking industry. The theoretical relationships between the variables is depicted as a theoretical model in Figure 1.

## Materials and Methods

A quantitative research methodology was deemed the most appropriate approach *via* a standardised quantitative questionnaire where participant responses were gathered in a web-based survey. The population selected for the purposes of the study consisted of 383 employees within the IT division of a South African retail bank. Probability sampling was applied as a sampling technique with participants selected by the researcher based on their proximity. The final sample consisted of 239 (*n*) individuals providing a 62.40% response rate to the questionnaire. The profile of the sample population in terms of biographical and demographic information is presented in Table 1.

**TABLE 1:** Biographical and demographic profile of respondents (*n* = 239).

Characteristic	Frequency (*n*)	Percentage (%)
**Tenure in the organisation**		
Less than 1 year	50	20.9%
1–3 years	84	35.1%
4–7 years	56	23.4%
8–10 years	23	9.6%
Longer than 10 years	26	10.9%
**Employment type**		
Permanent	237	99.2%
Contract	2	0.8%
**Age**		
Younger than 21 years	7	2.9%
21–25 years	40	16.7%
26–29 years	33	13.8%
30–38 years	89	37.2%
39–45 years	35	14.6%
46–55 years	30	12.6%
Older than 55 years	5	2.1%
**Gender**		
Male	193	80.8%
Female	46	19.2%
**Ethnicity**		
African/Black	14	5.9%
Coloured	68	28.5%
Indian/Asian	6	2.5%
White	151	63.2%

### Measuring Instruments

The first section of the survey consisted of gathering of the participants’ biographical and employment information. The subsequent sections provided a measurement of the specific latent variables using valid and reliable measuring instruments, including the Utrecht Work Engagement Scale (UWES*-*17) designed by Schaufeli et al. (2002), the Job Demands-Resources Scale (Jackson and Rothmann, 2005), and Roodt’s (2004) Turnover Intentions Scale (TIS).

The UWES-17 is a self-report questionnaire that consists of 17 items, measuring the three underlying dimensions of work engagement, including vigour (six items), dedication (five items), and absorption (six items) (Schaufeli and Bakker, 2003) scored on a seven-point Likert scale with varying poles of intensity ranging from 0 (never) and 6 (always). The UWES-17 has also been validated in several countries, including during South African studies conducted by Schaufeli and Bakker (2003) and Storm and Rothmann (2003).

The JDRS was used to measure job demands and job resources of employees, consisting of 48 items rated on a four-point Likert scale ranging from 1 (never) to 4 (always). According to Jackson and Rothmann (2005), the dimensions of JDRS consist of seven reliable factors, including organisational support (α = 0.88), growth opportunities (α = 0.80), overload (α = 0.75), job insecurity (α = 0.90), relationship with colleagues (α = 0.76), control (α = 0.71), and rewards (α = 0.78). As Rothmann and Jordaan’s (2006) study found highly acceptable alpha coefficients (ranging from 0.76 to 0.92), the scale indicates acceptable internal consistency reliability.

Turnover Intentions Scale was applied to measure the probability that employees of the IT division within a retail bank would quit their job in the foreseeable future. The TIS consists of 15 items that are measured on a five-point Likert response scale ranging from 1 (never) to 5 (always). Two earlier studies verified Roodt’s (2004) questionnaire as both reliable and factually valid. Jacobs (2005) reported a Cronbach’s alpha coefficient of 0.91 for the 15-item version of the TIS. Martin (2007) and Martin and Roodt (2008) reported a Cronbach’s alpha coefficient of 0.90 for a 13-item version of the scale. In addition, Du Plooy and Roodt (2010) reported a Cronbach’s alpha coefficient of 0.80. Both factor and reliability analysis was carried out during data analysis to determine the instrument’s reliability and validity on the specific study sample.

### Statistical Analysis

The data transformation process consisted of three broad phases. During the first phase, the proposed structure and reliability of the utilised JDRS, UWES and TIS measuring instruments were revalidated for the study sample through the application of confirmatory factor analysis (CFA). In the cases where poor fit of the data to the original conceptualisation of the instrument was found, exploratory factor analysis (EFA) was conducted. The maximum likelihood estimator with promax rotation was used to estimate the EFA models. SPSS version 26 (IBM Corporation, 2019) was used to conduct the EFA analyses.

In order to evaluate the CFA models, MPLUS 6 (Muthén and Muthén, 2010) was utilised. The goodness-of-fit statistics as well as model parameters were evaluated to determine the construct validity of the measures. The fit of the models were assessed with the Satorra-Bentler Chi-square (χ2), the root mean square error of approximation (RMSEA), the comparative fit index (CFI), and the Tucker–Lewis Index (TLI). Following Hu and Bentler’s (1999) guidelines, values of 0.90 for the CFI and TLI were deemed acceptable, whereas values of 0.95 or higher were considered indicative of excellent fit. For the RMSEA values, up to 0.08 represented reasonable errors of approximation (Browne and Crudeck, 1993).

Substantive hypotheses were empirically corroborated when the direction, magnitude, and statistical significance of path coefficients were congruent with *a priori* theorising. Ideally standardised factor loading estimates should be at least 0.50, but optimally 0.70 or higher. However, standardised loading estimates of 0.30 are still acceptable as an absolute minimum normative boundary, according to Du Plessis (2014).

Reliability analysis using Cronbach’s alpha (α) was also performed to determine whether the newly structured measurement instruments would produce consistent results with continued application. A minimum acceptable value of 0.70 was used in the current study (Nunnally, 1978). Descriptive statistics [including the mean (*M*), standard deviation (*SD*), skewness, and kurtosis] were provided during the second phase of the data analyses. The final analysis phase attempted to conceptualise and test the proposed structural model including the direct effects, indirect effects and moderating effects.

### Ethical Considerations

During the selection of the most appropriate questionnaires, care was taken to include questionnaire items free from potential bias. Informed consent was incorporated into the research design and data collection process, ensuring that individuals voluntarily participated in the research with the full knowledge of the purpose of the study, how the data will be analysed and reported on, and who the relevant internal and/or external parties are that will have access to the information. Participants were requested to complete the questionnaire voluntarily and assured that they can withdraw from the research at any time. The questionnaire was accessed *via* an anonymous on-line link, and limited biographical information was requested for reporting purposes. For participants choosing to complete a paper-based questionnaire (i.e., due to limited access to a computer), a centrally located sealed box was made available for the delivery of the completed questionnaire at a time convenient for the participant. Ethical clearance was received from the researchers’ university.

## Results

### Validity and Reliability of the Measurement Models

Both confirmatory and exploratory factor analyses were conducted on the individual measures to make sure that they demonstrate a basic level of construct validity. The results of the structural model will remain ambiguous if the integrity of the measurement models is not established initially (Kline, 2011). In the process of investigating the construct validity of the individual measures making up the structural model the authors considered the model fit indices, model parameters, and residuals. The descriptive statistics of the revised measures will be presented in the next section.

Some measurement inadequacies were identified in some of the original scale items. In an attempt to reduce the amount of variance in the measurement models that is attributable to random error variance, some items were deleted from the original measures. Initially a CFA model was fitted on all the individual measurement models. Only when the measurement models failed to fit the data was EFA used to explore possible areas of model strain or misfit.

In the Job Demands Model item 10 were deleted due to a low standardised factor loading of 0.181. Most of the items reported strong standardised factor loadings in excess of 0.50. However, a large number of modification indices indicated that not all items may be loading on a single factor. This may indicate that the Job Demands model is not uni-dimensional. As a result, the authors decided to specify an unrestricted EFA model. The EFA analyses suggested a three factor structure as outlined below.

• Factor 1 comprises four items (JDRS1 to 4) from the original overload dimension. These items focus specifically on the amount of work and time pressure associated with completing work, this factor is therefore renamed to **workload.**
• Factor 2 comprises three items (JDRS 7 to 9) from the original overload dimension. As these items speak to the personal and mental challenges people are confronted with during the performance of their work, this factor will be renamed to **emotional load.**
• Factor 3 comprises two items (JDRS5 to 6) from the original overload dimension. The items loading on this factor relate to having to be attentive to many things at the same time (amount of work), this factor will remain **mental load.**

Based on this configuration, a second order model was specified with three factors. The factor loadings for the latent constructs ranged between 0.38 and 0.74, and 88% of the standardised factors loadings were greater than 0.65. The model also indicated acceptable fit with regard to RMSEA (0.025), CFI (0.992), TLI (0.988), and SRMR (0.035). In addition, all the first order factors reported strong standardised factor loadings with the second order factor (0.143–0.643). Considered collectively, the second-order specification of the Job Demands Model fits the empirical data satisfactorily.

Job Resources consisted of Growth Opportunities, Organisational Support, Job Security, and Advancement. Since most of the items constituting each of the dimensions are uni-dimensional the authors decided to fit a second order model to the data. All the items reported strong standardised factor loadings to their respective first-order factors except items 47 and 48 belonging to the Advancement dimension. It was decided to delete these items because they reported low standardised factor loadings. The final higher order model reported good fit statistics with regard to RMSEA (0.090), however, the CFI (0.767), TLI (0.751), and SRMR (0.079) indices indicated that the model did not fit the data well. However, all the first order factors reported strong standardised factor loadings with their respective factors (0.456–0.976) and low correlated residuals. Although the fit of the model to the data cannot be regarded as statisfactory, the second-order CFA model fitted the data reasonably well and the decision was made not to revise the model further.

The Work Engagement measure consists of three dimensions, namely, Vigour, Dedication, and Absorption. Each of the dimensions are developed to be unidimensional. However, the authors did not formulate hypotheses specific to each of these three dimensions and as a result, it was decided to specify a higher-order CFA model to the data. In general, the standardised factor loadings were all in excess of the lower boundary threshold of 0.50 and the residuals were small and uncorrelated. The model fit also suggested that the three factor second-order CFA model fits the data adequately with the RMSEA (0.090), CFI (0.882), TLI (0.861), and SRMR (0.072) reporting adequate fit. Although the CFI and TLI did not reach the normative cut-off value of 0.90, both values were only marginally lower than 0.90. Considered collectively the authors were satisfied that the hierarchical CFA configuration of Work Engagement demonstrated sufficient construct validity to be included in the structural model.

Finally, the Turnover Intention measure was specified with a CFA model. The initial single factor CFA model did not fit the data well and it was decided to delete item 11 because it has a low standardised factor loading of 0.017. All the other items reported standardised factor loadings greater than 0.30 and most of the items reported factor loadings bigger than 0.70. Although the model fit improved with the deletion of item 11, the fit can still not be regarded as satisfactory since most of the fit indices did not fall within acceptable cut-off scores (RMSEA = 0.124; *p* > 0.05; CFI = 0.801; TLI = 0.764; SRMR = 0.073) (Hu and Bentler, 1999). The high RMSEA value was particularly concerning since the confidence interval was quite wide and the point estimate high. Considered collectively, the Turnover Intention measurement model demonstrated mediocre fit to the data. However, in trying to diagnose possible areas of misfit, the authors felt it was imprudent to correlate residuals or to delete any further items in order to artificially improve the model fit.

The means, standard deviations, correlations, and Cronbach’s Alpha reliability scores for each of the sub-dimensions are summarised in Table 2.

**TABLE 2:** Means, standard deviations, Cronbach’s alpha values, and correlations between factors.

	Variable	*M*	*SD*	*Alpha*	*1*	*2*	*3*	*4*	*5*	*6*	*7*	*8*	*9*	*10*
1	JDRS1_Workload	**2.92**	**0.54**	**0.70**										
2	JDRS2_Emotional load	**1.97**	**0.59**	**0.74**	0.26**									
3	JDRS3_Mental load	**3.41**	**0.61**	**0.65**	0.44**	0.12								
4	JDRS_Growth	**2.81**	**0.59**	**0.87**	0.24**	−0.22**	0.28**							
5	JDRS_Support	**3.04**	**0.53**	**0.94**	0.07	−0.36**	0.19**	0.68**						
6	JDRS_Job security	**2.12**	**0.95**	**0.92**	–0.03	–0.02	0.14*	0.12	0.12					
7	JDRS_Advancement	**2.41**	**0.73**	**0.88**	0.12	−0.15*	0.09	0.42**	0.33**	–0.07				
8	UWES_Vigour	**4.00**	**0.97**	**0.86**	0.24**	−0.16*	0.30**	0.53**	0.53**	0.03	0.26**			
9	UWES_Dedication	**4.30**	**1.16**	**0.90**	0.19**	−0.25**	0.25**	0.72**	0.64**	0.18**	0.38**	0.70**		
10	UWES_Absorption	**3.79**	**0.92**	**0.79**	0.23**	0.03	0.18**	0.43**	0.30**	0.02	0.33**	0.57**	0.59**	
11	TIS	**2.42**	**0.754**	**0.90**	–0.02	0.44**	–0.04	−0.57**	−0.54**	0.14*	−0.54**	−0.56**	−0.65**	−0.43**

JDRS, job demands resources scale; UWES, Utrecht Work Engagement Scale; TIS, Turnover Intentions Scale; M, mean; SD, standards deviation; **, significant at the 0.01 level; *, significant at the 0.05 level.

Results from Table 2 suggest that most standard deviation scores were in the same range except for the dedication dimensions of work engagement which reported a higher standard deviation score. Most of the Cronbach’s alpha values were also higher than 0.70 except for the mental load dimension of Job Demands.

### Assessment of the Structural Models

The primary objective of the study was to empirically validate the proposed structural model. The core psychological mechanism in the model propose an interaction between job demands and job resources which have an impact on turnover intentions through work engagement. As such the model included direct effects, indirect effects, and interaction effects. Testing for interaction effects and indirect effects typically require high statistical power and often different statistical estimators (Muthén and Muthén, 2010). One can specify a multi-group model to see if the model differs across different levels of the moderator but the authors opted for a strategy to test multiple models. The benefit of this approach is that the impact of the moderators and mediators can be examined individually. A notable disadvantage is that the entire proposed model cannot be assessed in one single step.

Our approach was to test the proposed structural model portrayed as Figure 1 initially including all direct and indirect effects. We used robust Maximum Likelihood (RML) to test this model and refer to this model as the mediation model. Second, we tested the model depicted in Figure 1 with the interaction term, but without specifying the indirect effect. For this model, we used the integration algorithm and this model was referred to as the interaction model. Although we did not test the direct, indirect and interaction terms in a single model, the parameters that were consistent across the two models remained relatively stable in terms of path coefficients, which instilled confidence in the generalisability of the parameters across the two models.

The fit indices of the mediation and interaction models are summarised in Table 3.

**TABLE 3:** Fit indices for the mediation and interaction models.

Fit indices	*Mediation model*	*Interaction model*
**Absolute fit**		
LogLikelihood H0 value	20684.101	20681.842
Degrees of freedom	247	248
Scaling correction factor for Robust ML	1.0439	–
RMSEA (Root mean square error of approximation)	0.067	–
*P*-value RMSEA (≤0.05)	0.001	–
90% confidence intervals	0.064; 0.069	–
Standardised root mean squared residual (RMR)	0.099	–
**Incremental fit**		
Comparative fit index (CFI)	0.725	–
Tucker–Lewis fit index (TLI)	0.716	–

When using the integration estimator to estimate interaction effect not all the conventional fit indices are provided. However, residuals can still be examined for interaction model to determine the relative fit of the model to the data. With regard to the interaction model all the structural (zeta) and items residuals (theta delta) were relatively small, except for job security (θ_δ_ = 0.0985) and advancement (θ_δ=_ 0.734). This would suggest that most of the common variance in the items are explained by the latent dimensions and not error. This is unfortunately not true with regards to the job security and advancement dimensions. However, all the other dimensions reported relatively low residual values.

When looking at the fit of the mediation model only the RMSEA value (0.076) was indicative of good model fit. The other fit indices including the CFI (0.725), TLI (0.716), and SRMR (0.099) indices indicated that the model did not fit the data well. The fit indices of the two models suggest that the overall model fit can be regarded as mediocre.

Turning to the specific model parameters, Table 4 contains the structural regression parameters with associated standard errors and *p*-values. The model parameters are presented for both the mediation and interaction models.

**TABLE 4:** Standardised regression weights in the mediation and interaction structural models.

	*Mediation model*			*Interaction model*		
Hypotheses	β	B	S.E.	β	B	S.E.
H_1_: JR → WE	0.845	1.419***	0.039	0.845	1.413***	0.185
H_2_: JD → WE	0.055	0.124	0.055	0.054	0.130	0.166
H_3_: JRxJD → WE	−	−	-	–0.100	−0.449*	0.048
H_4_: JR → TI	–0.409	−0.711*	0.190	–0.373	–0.651	0.372
H_5_: JD → TI	0.270	0.634**	0.068	0.267	0.666**	0.199
H_6_: WE → TI	–0.468	−0.484**	0.180	–0.506	−0.529**	0.201
H_7_: JR → WE → TI	−0.395**	−0.687**	0.237	−	−	-

*JR, job resources; JD, job demands; WE, work engagement; TI, turnover intentions; B, unstandardised path coefficient; S.E., standard error; β, standardised path coefficient.*

*p < 0.05; **p < 0.01; ***p < 0.001.

In line with theorizing, in both models job resources were positively related to work engagement (β = 0.845; *p* < 0.001), yet statistical support was not found for the proposed relationship between job demands and work engagement. Furthermore, support was found for the moderating role of job demands on the relationship between job resources and work engagement (β = −0.100; *p* < 0.05). Thus, job demands seem to have a significant interaction effect with job resources in relation to work engagement. Statistical support was found for the proposed relationship between job resources and turnover intentions but only in the mediation model (β = 0.711; *p* < 0.05) and not the interaction model. However, strong support was found for the proposed relationship between job demands and turnover intention in both the mediation (β = 0.634; *p* < 0.05) and interaction models (β = 0.666; *p* < 0.01).

Statistical support was also found for the proposed relationship between work engagement and turnover intention in both the mediation (β = −0.484; *p* < 0.01) and interaction (β = −0.529; *p* < 0.01) models. Finally, support was found for the indirect effect between job resources and turnover intentions *via* work engagement (β = −0.395; *p* < 0.01). The results suggest that support was found for most of the proposed direct, indirect and interaction relationships in the proposed model. The results will be discussed in the next section.

## Discussion

The central aim of this study was to explore the impact of job resources and job demands on work engagement and employee turnover intentions within the IT division of a South African bank. The summary of the findings provided in the following section could serve as suggested guidelines to organisations within the financial services and banking industries during the development of a retention strategy to increase employee work engagement and intention to stay.

### Interpreting the Appropriateness of the Selected Measurement Model

The Job Demands-Resources Scale (JDRS) conformed to a seven-factor model with four dimensions belonging to the job resources category and three unique job demands dimensions loading onto any of the factors in the new measurement model during the factor analysis process.

The research population associated items within the job demands dimension of the original job demands scale with three distinct dimensions, defined as **workload** (α = 0.70, 4 items), **emotional load** (α = 0.74, three items), and **mental load** (α = 0.65, two items). De Braine and Roodt (2011) grouped job demands into quantitative job demands (i.e., time pressure, work overload) and qualitative job demands (i.e., emotional demands, role ambiguity, role conflict, and unfavourable physical work environment). The first distinct factor is identified was defined as “overload” and includes physical, cognitive and emotional load related to time pressure (pace of work), attentiveness to many things at the same time (amount of work), and mental and emotional load (dealing with power struggles). Depending on the job context, Van den Broeck et al. (2008) are of the opinion that the category of job demands can contain job characteristics as varied as task interruptions, workload, work-home interferences, organisational changes, and emotional dissonance (e.g., Bakker et al., 2003b).

The data still supports **growth opportunities** (α = 0.87, eight items), **organisational support** (α = 0.94, twenty one items), **advancement** (α = 0.88, four items), and **job security** (α = 0.92, three items) to remain unique dimensions allocated to the job resources scale. The second order CFA suggests that growth opportunities are the strongest job resource, followed by organisational support and advancement. The indicated degree of importance attached to the identified job resources should be a key consideration for companies when developing and prioritisation the key focus areas within a robust retention strategy.

The results of exploratory factor analysis provided sufficient statistical support for the existing three-factor work engagement model, including vigour (α = 0.86, 6 items), dedication (α = 0.90, 4 items), and absorption (α = 0.79, 6 items). Research conducted by Schaufeli and Bakker (2004) and Barkhuizen and Rothmann (2006) reported acceptable Cronbach’s alpha internal consistency reliability coefficients for the three subscales, ranging between 0.68 and 0.91. The applicability of the original three dimension Utrecht Work Engagement Scale (UWES-17) to a South African sample has been validated in previous studies (i.e., Schaufeli and Bakker, 2003; Storm and Rothmann, 2003).

The Turnover Intentions Scale (TIS) was also confirmed as a one-dimensional construct after the removal of 1 item (α = 0.90, 14 items). The turnover literature lacks formally validated scales to represent turnover cognitions (Sager et al., 1998). Various researchers have used only one item (Guimaraes, 1997; Lambert et al., 2001). The approach to use single-item indicators to measure turnover cognitions is criticized since they typically lack reliability (Sager et al., 1998; Lee et al., 2000). Only a few studies could be found where more than three items per instrument were used (Lum et al., 1998; Fox and Fallon, 2003), which informed our choice to use the 14-item TIS.

### Interpreting the Findings of the Research Hypothesis

The results of the structural equations model indicated a statistically significant path from job resources to work engagement (β = 0.845; *p* < 0.001). The finding that job resources have a significant impact on work engagement are in line with the conservation of resources (COR) theory (Hobfoll, 1989) which stated that if organisations fail to provide sufficient job resources (i.e., growth opportunities, role clarity, social support, and financial rewards), employees will start exhibiting withdrawal behaviour from work, including a decline in motivation and commitment (Hobfoll, 1989). According to Coetzer (2006), the availability of growth opportunities play a critical role in enhancing work engagement. Level of work engagement is further impacted by the availability of career growth opportunities through clear career paths and development opportunities (Mehta and Mehta, 2013). According to Rothmann and Pieterse (2007), work engagement (including vigour and dedication) is exclusively predicted by the availability of opportunities for job growth and the experience of a strong sense of coherence. Employees will exhibit higher levels of dedication and vigour at work when perceiving that they have access to opportunities to learn, variety in their work, and a level of independence in the execution of their tasks. Mehta and Mehta (2013) also highlighted the importance of the social support provided by colleagues and the quality of relationship between team members as a driver of engagement, characterised by mutual respect, feelings of being part of an efficient team, and having a good relationship with work colleagues, according to Robinson et al. (2004). Bakker and Demerouti (2007) reported that supportive colleagues and suitable supervisory feedback contribute to an increased likelihood of employees successfully achieving their work goals, and satisfies the employee’s need to belong (Schaufeli and Bakker, 2004). According to Chen and Silverthorne (2005), leadership behaviours have a strong influence on the employee and organisational outcomes, including work engagement.

On the converse, the hypothesised relationship between job demands and work engagement was not statistically significant (β = 0.055). According to Bakker and Demerouti (2007), the JD-R model theoretically does not assume any direct association between job demands and work engagement. Previous research has, however, investigated the potential impact of job demands on the levels of work engagement in employees. Empirical studies based on the JD-R model have shown that some types of job demands (i.e., workload, time pressure, cognitive demands, etc.) are positively associated with work engagement both concurrently (LePine et al., 2005) and over time (Mauno et al., 2007). Conversely, Mauno et al. (2007) and Prieto et al. (2008) found that different types of job demands (i.e., role ambiguity, role conflict, etc.) are negatively associated with the dedication component of work engagement over time.

In support of hypothesis 3, job demands did, however, have a moderating role on the relationship between job resources and work engagement (β = -0.100; *p* < 0.05). Existing literature has made reference to the moderating impact of job demands on the relationship between job resources and work engagement. In Bakker and Demerouti’s (2007) overall model of work engagement, it is described that job demands moderate the resources-engagement relationship. It is a central assumption within the JD-R model that job resources become more significant and gain motivational potential when employees are confronted with high job demands (i.e., Bakker and Demerouti, 2007). A study by Hakanen et al. (2005) indicated that job resources are more beneficial in maintaining the level of work engagement under conditions of high job demands. Similar findings were also reported by Bakker et al. (2008) in a study among Finnish teachers that found job resources buffer and diminish the negative relationship between pupil misbehaviour (job demand) and work engagement. Additionally, it was reported that job resources had a particularly significant influence on the work engagement levels of teachers when they were confronted with high levels of misconduct (job demand).

The study results indicate that this specific sample of IT employees will attach significance to the availability of job resources when deciding to remain or leave their employer (β = −0.409). Job resources are considered to be crucial for employee retention, according to De Braine and Roodt (2011). Various studies (i.e., Demerouti et al., 2001; Bakker et al., 2004; Schaufeli and Bakker, 2004; Bakker and Demerouti, 2007) indicated the absence of job resources is related to disengagement, which in turn, increases turnover intentions. The availability of specific job resources will offer a more salient buffer against turnover intentions in IT employees. Furthermore, if an organisation provides resources that enable the employee to perform his or her duties successfully, the employee may be hesitant about leaving the organisation (Halbesleben and Wheeler, 2008). Thus, hypothesis 4 was accepted.

The relationship between job demands and turnover intentions has was statistically significant (β = 0.270^**^) as outlined in hypothesis 5. Job and organisational design literature have revealed various job demands that are positively related to turnover intentions, including disproportionate workload (i.e., Houkes et al., 2003), role stressors associated with performing tasks not in the employee’s job description or role ambiguity (i.e., Asiwe et al., 2015), and a lack of challenge (i.e., Mathieu and Zajac, 1990) characterised by task repetitiveness and excessive routine (i.e., Griffeth et al., 2000). According to Asiwe et al. (2015), excessive workload or high demands may also occur when an individual does not have the required skills, abilities and support to meet the expressed demands. High levels of stress are, therefore, prevalent in individuals experiencing work overload (Schaufeli and Bakker, 2004), characterised by feeling overwhelmed by perceived time pressures and deadlines, excessive work demands and information overload (Montgomery et al., 2003). Hoonakker et al. (2013) are also of the opinion that work-family conflict could be considered a job demand, and has been negatively linked to various organisational outcomes, including job satisfaction, organisational commitment, job stress, and turnover (Ahuja, 2002; Hoonakker et al., 2005). As many IT related jobs expect employees to work late, be on-call to address technical problems, and even travel extensively, all of these factors can contribute to conflict between work and family-life.

The statistically significant negative relationship between work engagement and turnover intentions was found (β = −0.468) in support of hypothesis 6. Schaufeli and Bakker (2004) stated that engaged employees are likely to have a greater attachment to their organisation and a lower tendency to quit. According to Mendes and Stander (2011), engaged employees exhibit an awareness of the organisational context in which they operate, and will work with others to improve their performance within their roles to the benefit of the organisation (Devi, 2009). These findings are further supported by Baskin (2007) who stated that employees experiencing low engagement levels are more likely to leave an organisation. As employees operating within the IT division of this specific South African retail bank become more engaged in their work, they will be less prone to seek alternative opportunities outside of their current employer. Thus, hypothesis 6 was accepted.

Finally, hypothesis 7 suggested that work engagement mediates the relationship between job resources and turnover intentions. Support was found for the indirect effect between job resources and turnover intentions *via* work engagement (β = 0.395; *p* < 0.01). This suggests that job resources can have a direct influence on the employees’ intentions to leave, with or without the interaction of work engagement. Although Schaufeli and Bakker (2004) found evidence for work engagement as full mediator of the relationship between job resources and turnover intentions, the current research only found evidence of work engagement as partial mediator. The availability of suitable job resources can influence the turnover intentions of the IT employees directly and indirectly *via* work engagement as intermediate or mediator variable.

### Significance of the Study

The main objective of this current study was to gain insight into the specific job resources contributing to work engagement within the IT division of a South African bank, and to share these key learnings to influence the design of focused strategies to ensure continued retention of the organisation’s scarce and critical IT skills and resources. From the research findings, the need for organisations to provide employees with access to job resources associated with quality and in-depth **social support**, opportunities for continued growth and professional development, a high sense of **job security**, and opportunities for **advancement** within the organisation were highlighted. **Social support** is one of the most well-known situational variables proposed as potential buffer against job and environment related stressors (i.e., Haines et al., 1991). Management support should also include recognition and feedback given to employees on their performance (Kochanski and Ledford, 2001), ultimately leading to greater feelings of importance and level of responsibility towards the organisation due to the employee being offered an opportunity to use their innovation and skill to the advantage of the organisation, according to Eisenberger et al. (1990).

Previous between-person studies have also consistently shown that job resources, such as support from co-workers and supervisors, performance feedback, autonomy, and opportunities for **professional development**, are positively associated with work engagement. Furthermore, Bosman et al. (2004) were of the opinion that increased **job insecurity (as a stressor)** will be associated with increased levels of burnout and decreased levels of work engagement. As work engagement is considered a significant factor under conditions of great uncertainty (Macey and Schneider, 2008), it is expected that highly engaged employees will exhibit a stronger need to alter or change the task and relational boundaries of their jobs in environments with high job insecurity in an attempt to reduce uncertainty and to provide a better fit with their specific values and needs, according to Lui et al. (2014). In this study, access to **advancement opportunities** had a significant impact on the turnover intentions of the IT employees. If the employee perceives advancement opportunities within their current organisation as lacking when compared with other organisations within the same industry, feelings of comparative deprivation may be triggered, according to Alhamwan and Mat (2015), increasing turnover intentions or actual turnover among employees (i.e., Heslin, 2005; Zhao and Zhou, 2008).

The current study also highlighted that IT resources does not view job demands as uni-dimensional, but that a combination of work, emotional and mental load contribute to levels of work engagement and intention to stay. Maslach et al. (1996) hypothesized that the presence of specific demands (i.e., work overload and personal conflicts) and the absence of specific resources (i.e., control coping, social support, autonomy, and decision involvement) predicts burnout which, in turn, is expected to lead to various negative outcomes, including an increase in employee turnover. The pro-active and effective management of work engagement and turnover intentions would require organisations to follow a strategic approach, according to James and Mathew (2012), requiring a continuous diagnosing of the antecedents of work engagement and turnover intentions, supported by the development of a targeted and well-structured retention approach (Allen et al., 2010).

### Study Limitations and Recommendations for Future Research

The research data was gathered in a cross-sectional sample from a single retail bank, hence the question of generalisability of results and identified trends to the IT divisions of other financial institutions (especially banks) could still be questioned. Data collection was also done *via* a self-administered web-based survey that could be prejudiced by social desirability when participants decide to respond to the questionnaire in a manner that could lead to the creation of a more favourable impression of themselves (Podsakoff et al., 2003).

The JD-R model (Demerouti et al., 2001; Bakker and Demerouti, 2007) was applied as theoretical framework for the study of work engagement, assuming every work environment has unique characteristics that can be captured in one overall model. A potential limitation of the JD-R model is its exclusive focus on the psychosocial work environment by defining job demands and job resources only in terms of the positively and negatively valued work characteristics. It is recommended that future research extend the application of the JD-R model to include factors not related to work, including the potential impact of personal resources and employees’ application of job crafting behaviour to shape a job in accordance with the individual’s preferences, skills and abilities (Tims and Bakker, 2010; Thomas et al., 2020). Furthermore, the use of cross-sectional data to test the proposed relationships within the JD-R model cannot provide evidence of the temporal order of the variables, or the reverse and reciprocal causal relationships (Lesener et al., 2019). Although various South African studies have reported support for the original JD-R model (Demerouti et al., 2001; Schaufeli and Bakker, 2004; Bakker and Demerouti, 2007), Rothmann et al. (2006) emphasised the importance of more research required to develop a valid measure applicable to a wide variety of contexts.

## Summary and Conclusion

By exploring the specific factors that contribute to the occurrence of work engagement and turnover intentions amongst employees within the IT division of a South African bank, this study made a positive contribution to the theoretical framework of work engagement and turnover intention. To summarise the following was found:

• Job resources (specifically social support, job security, and advancement opportunities) are key to foster improved work engagement and retention of IT professionals.
• Job demands should be managed carefully, as excessive job demands may lead to higher levels of staff turnover, and decrease the usefulness of job resources in fostering work engagement.
• Work engagement of IT professionals can counteract intentions to quit, thus informing retention strategies in an industry known to have high staff turnover.

Although the findings of the current study are based on data gathered within a single organisation, the results obtained do provide encouraging deductions on the specific job resources impacting work engagement and intention to stay of IT employees within the South African banking industry. By applying the JD-R model as a theoretical framework for the study, the unique job resources and job demands as drivers of work engagement and turnover intentions of IT employees could be highlighted to direct the development of focused work engagement and retention strategies. It is hoped that this research will add value to organisational knowledge on how to improve work engagement and intention to stay of scarce and critical IT skills within this highly competitive industry.

## Data Availability Statement

The raw data supporting the conclusions of this article will be made available by the authors, without undue reservation.

## Ethics Statement

The studies involving human participants were reviewed and approved by Senate Research Committee of the University of the Western Cape. The patients/participants provided their written informed consent to participate in this study.

## Author Contributions

This manuscript was an outflow of the thesis by JV who conceptualised the study and collected all the data. MD was the supervisor of the study. JB assisted with the statistical analysis. All authors worked collaboratively on the draft manuscript, contributed to the article, and approved the submitted version.

## Conflict of Interest

The authors declare that the research was conducted in the absence of any commercial or financial relationships that could be construed as a potential conflict of interest.

## Publisher’s Note

All claims expressed in this article are solely those of the authors and do not necessarily represent those of their affiliated organizations, or those of the publisher, the editors and the reviewers. Any product that may be evaluated in this article, or claim that may be made by its manufacturer, is not guaranteed or endorsed by the publisher.
